# Organic Semiconductor Single Crystals: From Continuous Fabrication to Optoelectronic Applications

**DOI:** 10.3390/ma19194207

**Published:** 2026-10-02

**Authors:** Zhixiang Zhong, Jie Ren, Hanying Li

**Affiliations:** 1 MOE Key Laboratory of Macromolecular Synthesis and Functionalization, International Research Center for X Polymers, ZJU-YST Joint Research Center for Fundamental Science, Department of Polymer Science and Engineering, Zhejiang University, Hangzhou 310027, China; zhongzhixiang@zju.edu.cn; 2 Department of Physics, Chemistry and Biology (IFM), Linköping University, 58183 Linköping, Sweden

**Keywords:** organic semiconductor single crystals, crystallization kinetics, continuous fabrication techniques, charge transport mechanisms, electronic and optoelectronic devices

## Abstract

Organic semiconductors serve as the cornerstone for next-generation flexible, cost-effective, and large-area optoelectronics, offering profound research significance and immense industrial potential. Compared to their polycrystalline and amorphous counterparts, organic single crystals possess distinctive advantages, including long-range molecular order, minimal defect density, superior intrinsic carrier mobility, and robust environmental stability. Consequently, they provide an ideal benchmark for probing fundamental charge transport physics and developing high-performance devices. In this review, we briefly trace the evolution of organic semiconductor single crystals (OSSCs) before discussing recent breakthroughs in the field. We place particular emphasis on the crystallization kinetics, continuous fabrication techniques, carrier transport mechanisms, electronic and optoelectronic applications of OSSCs. Finally, we offer insights into future trajectories, focusing on crystallization theories, charge transport mechanisms, and device technologies compatible with scalable processing.

## 1. Introduction

Semiconductors are materials with electrical conductivities intermediate between those of conductors and insulators. Since the advent of semiconductor-based transistors in the last century, transistor technologies have driven the rapid development of modern information technology and the microelectronics industry, profoundly transforming modern society. Compared with conventional inorganic semiconductors, organic semiconductors offer several distinctive advantages, including low-temperature processing, low-cost manufacturing and mechanical flexibility. Moreover, the molecular structures of organic semiconductors can be readily tailored through molecular design, enabling the integration of diverse functionalities and properties and thereby providing broad opportunities for the development of organic optoelectronic devices. To date, organic semiconductors have been widely employed in a variety of electronic and optoelectronic devices, including organic field-effect transistors (OFETs), organic light-emitting diodes (OLEDs), and organic photovoltaics (OPVs) [1,2,3]. However, the optoelectronic properties of organic semiconductors are governed not only by their molecular structures but also by their solid-state organization. Different polymorphs or molecular packing arrangements can exhibit markedly different optoelectronic characteristics. For example, amorphous and polycrystalline organic semiconductor films generally exhibit disordered molecular orientations and contain abundant grain boundaries and structural defects [4]. Their macroscopic properties therefore arise from the combined effects of intrinsic molecular characteristics and structural disorder. Such disorder not only compromises key optoelectronic properties, such as charge-carrier mobility and exciton diffusion length [5], but also complicates the establishment of clear relationships between molecular structure and optoelectronic performance.

In contrast, organic semiconductor single crystals possess long-range structural order, well-defined crystallographic orientations, and substantially reduced densities of grain boundaries and structural defects. These characteristics minimize the influence of structural disorder and allow the intrinsic properties of organic semiconductors to be more directly elucidated, making single crystals ideal model systems for investigating their fundamental physical properties [6]. Accordingly, the significance of organic semiconductor single crystals extends beyond the pursuit of enhanced device performance. More importantly, they provide a well-defined platform for systematically uncovering the intrinsic relationships among molecular structure, molecular packing, and macroscopic optoelectronic properties. Such studies further provide an experimental foundation for establishing rational design principles for organic semiconductor materials.

As early as 1948, the semiconducting characteristics of metal-free phthalocyanine single crystals had already been recognized [7]. It was not until 1996, however, that Horowitz and co-workers reported the first field-effect transistor based on an organic single crystal [8]. In their study, α-sexithiophene single crystals grown by physical vapor transport were employed as the active semiconductor, yielding a field-effect mobility of 0.075 cm^2^ V^−1^ s^−1^. Since then, substantial progress has been made in improving the charge-carrier mobility of organic semiconductor single crystals. For example, Podzorov et al. [9] demonstrated p-type rubrene single-crystal field-effect transistors with mobilities of up to 8 cm^2^ V^−1^ s^−1^. Takeya and co-workers [10] subsequently improved the crystal/dielectric interface and increased the mobility to 18 cm^2^ V^−1^ s^−1^, while estimating the intrinsic hole mobility of rubrene to be as high as 40 cm^2^ V^−1^ s^−1^. Jurchescu et al. [11] further reported field-effect mobilities approaching 40 cm^2^ V^−1^ s^−1^ in pentacene single-crystal transistors. Such values exceed those typically obtained for amorphous silicon, which exhibits a mobility of approximately 1 cm^2^ V^−1^ s^−1^, and are sufficiently high for many practical electronic applications.

Despite these advances, high-quality organic semiconductor single crystals were initially prepared predominantly by methods such as physical vapor transport [12] and slow solvent evaporation [13]. These approaches generally provide limited control over crystal position, orientation, lateral dimensions, and thickness, making them poorly suited for device integration, array fabrication, and large-scale manufacturing. To overcome these limitations, a series of representative studies progressively advanced the controlled patterning, continuous growth, and scalable fabrication of organic single crystals. In 2011, Minemawari et al. [14] combined antisolvent crystallization with inkjet printing to achieve the direct deposition of C8-BTBT single-crystalline films, simultaneously enabling site-selective growth and high crystalline quality. This work was followed by the development of a variety of strategies for the large-area and continuous growth of organic semiconductor single crystals [15,16,17,18]. These advances in fabrication also highlighted that large-area single-crystal growth is governed by the coupled effects of multiple thermodynamic and kinetic processes. Accordingly, research on the morphological control of organic single crystals has gradually evolved from largely empirical optimization of processing parameters toward in situ interrogation [19] and theoretical description [20] of the crystallization process.

This review aims to provide an overview of the progress in organic semiconductor single crystals over the past several decades, with particular emphasis on the processing technologies and fundamental principles underlying their controlled fabrication, charge-transport mechanisms, and applications in electronic and optoelectronic devices. Section 2 focuses on strategies for the large-area and continuous fabrication of organic semiconductor single crystals. Benefiting from low-temperature processing, compatibility with diverse fabrication techniques, and the potential for scalable production, these approaches represent an important route toward continuous manufacturing and device-level integration of organic single crystals. Section 3 discusses the morphological control of organic single crystals, with particular attention to how various crystallization kinetic factors govern crystal growth and morphology. Section 4 addresses charge transport in organic semiconductor single crystals, examining the factors that influence carrier-transport behavior and the transport mechanisms, as well as their possible transitions, under different temperatures, applied electric fields, and interfacial environments. Finally, Section 5 summarizes recent advances in electronic and optoelectronic devices based on organic semiconductor single crystals, including their implementation in individual devices and integrated systems, and discusses the opportunities and challenges associated with achieving high-performance devices and large-scale integration.

## 2. Continuous Fabrication of OSSCs

The growth of organic semiconductor single crystals is a prerequisite for investigating their structures and intrinsic properties, as well as for constructing organic electronic devices. High-quality single crystals with minimal impurities and structural defects are essential for revealing the intrinsic characteristics of organic semiconductors and are also crucial for achieving high device performance. At present, the growth of organic semiconductor single crystals can be broadly classified into vapor-phase and solution-based methods. Vapor-phase methods, particularly physical vapor transport, are capable of producing high-purity bulk single crystals with high structural quality and crystal structures approaching the ideal limit. Consequently, many single crystals used in fundamental studies of molecular packing, crystal structure, and intrinsic physical properties have been prepared using this approach. For practical large-scale device fabrication, however, organic semiconductors are generally required to be in the form of thin films [21]. Random nucleation and island-like growth on substrates often lead to substantial variations in crystal thickness and orientation during vapor-phase deposition, making it difficult to obtain continuous and uniform single-crystalline films. Moreover, conventional epitaxial strategies widely employed for inorganic single-crystal thin films, which rely on lattice-matched substrates, are difficult to directly extend to organic semiconductors because suitable epitaxial substrates are generally unavailable [22]. As a result, vapor-phase methods remain challenging for the fabrication of large-area organic semiconductor single-crystalline films and are more commonly employed in fundamental studies involving individual crystals or discrete devices [23]. In addition, vapor-phase growth typically requires high-vacuum and elevated-temperature (such as 300 °C) conditions, resulting in relatively long processing times and high fabrication costs, while also imposing stringent requirements on the thermal stability of organic molecules.

In comparison, solution-based methods enable the growth of organic single crystals under ambient-pressure and low-temperature (such as room temperature) conditions, offering advantages such as operational simplicity and low processing cost. These characteristics make solution processing particularly attractive for scalable manufacturing and potential commercialization. Solution-based techniques have also been extensively employed in the fabrication of organic semiconductor thin films. With continuous improvements in processing strategies and the development of new crystallization methods, the crystalline quality of solution-processed organic semiconductor films, particularly in terms of crystalline domain size and molecular orientation, has been substantially improved, together with corresponding advances in device performance. This section therefore focuses on representative solution-processing strategies and their recent developments for the large-area and continuous fabrication of organic semiconductor single-crystalline films.

### 2.1. Droplet-Pinned Crystallization

**Figure 1:**
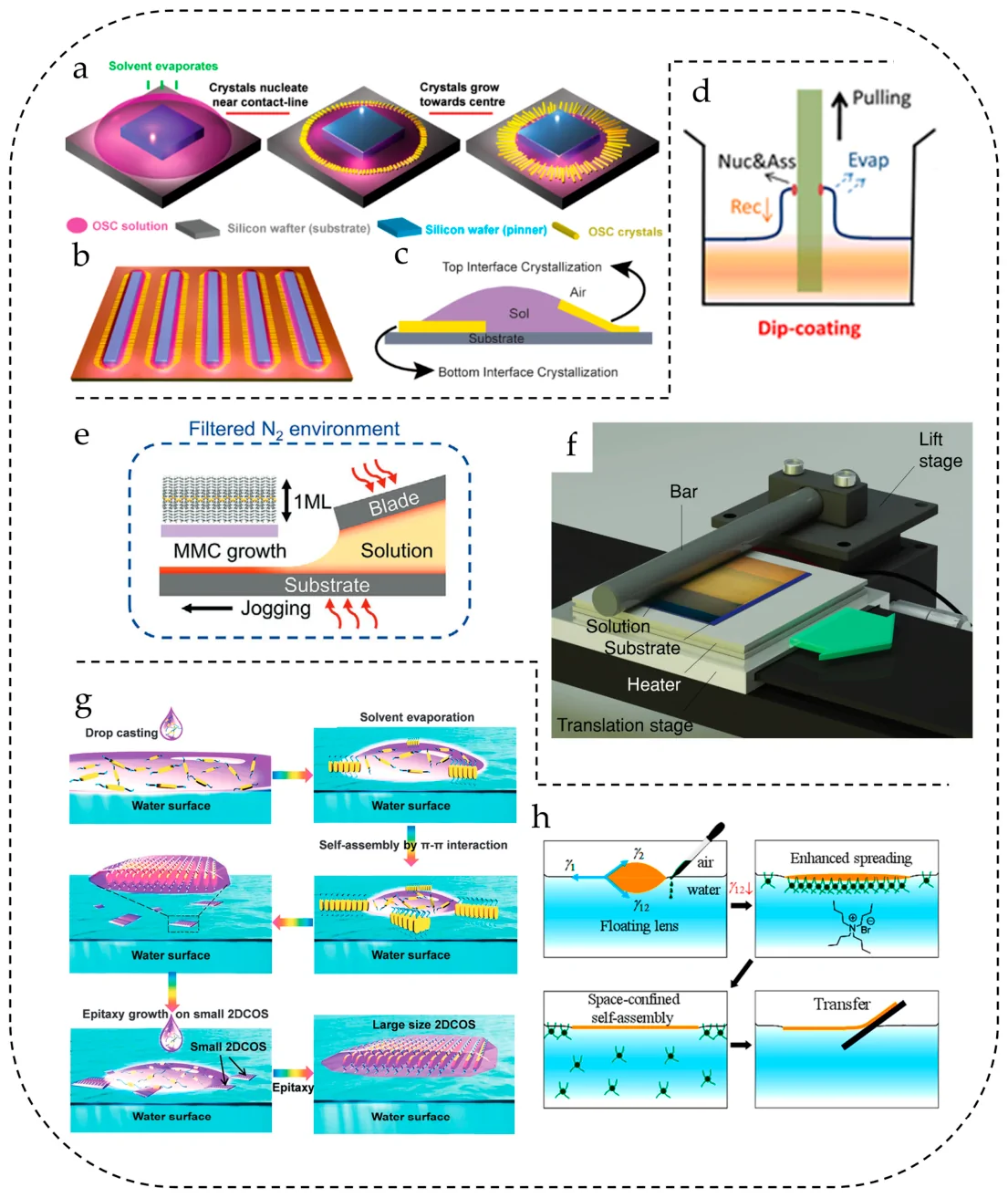
Large-area and continuous growth of organic semiconductor single crystals. (**a**–**c**) Schemes and morphologies of crystals grown using the DPC method [24]. (**a**) Schematic process for the growth of aligned single crystals. (**b**) Large-area growth of aligned single crystals using multiple pinners. (**c**) Schematic representation of crystal formation from either top- or bottom-interface crystallization. (**d**) Schematic illustration of the dynamic processes in dip-coating. Reproduced with permission [27]. Copyright 2016, American Chemical Society. (**e**) Chemical structure of C8-DNTT and schematic solution shearing process for the deposition of C8-DNTT single-domain molecular monolayer crystals. Reproduced with permission [17]. Copyright 2023, Wiley-VCH. (**f**) Schematic illustration of bar-coating. Reproduced with permission [28]. Copyright 2017, Wiley-VCH. (**g**) Schematic diagrams of growing 2D crystals of organic semiconductor by “Solution Epitaxy”. Reproduced with permission [18]. Copyright 2016, 2018 Wiley-VCH. (**h**) Sketches of the SCS method for the growth and transfer of 2D molecular crystal. Reproduced with permission [29]. Copyright 2018, American Chemical Society.

Drop casting is a particularly simple deposition method in which an organic semiconductor solution is deposited onto a target substrate and crystals precipitate as the solvent evaporates. During droplet shrinkage, however, the droplet can readily move across the substrate, resulting in unstable and nonuniform deposition. Consequently, conventional drop casting generally produces discrete small crystallites or polycrystalline films with disordered orientations. To address this limitation, Li et al. [15,24] proposed the droplet-pinned crystallization (DPC) method. As illustrated in Figure 1a, the basic principle of DPC is to anchor the droplet using a silicon wafer or another pinning element, such as a needle or capillary, thereby enabling stable recession of the substrate-solution-air three-phase contact line at the droplet edge. As the solvent evaporates, nuclei formed near the contact line can grow steadily and continuously along the direction of contact-line recession toward the droplet center, producing highly oriented arrays of ribbon-like single crystals. The ribbon crystals can extend over hundreds of micrometers and even reach millimeter-scale lengths [25]. Because charge carriers can travel along the long axis of the ribbons within a single crystalline domain, field-effect transistors based on ribbon-like TIPS-PEN and C_60_ single-crystal arrays exhibited average hole and electron mobilities of 2.4 and 3.0 cm^2^ V^−1^ s^−1^, respectively. Another important advantage of DPC is that the growth area can be readily enlarged by adjusting the shape, dimensions, and positions of the pinning elements. For example, as shown in Figure 1b, multiple elongated pinning elements enabled oriented crystal growth over a 4-inch wafer. Moreover, DPC can simultaneously grow two different organic semiconductor single crystals at distinct interfaces, allowing an organic single-crystal heterojunction to be formed in a single step from a mixed solution, as shown in Figure 1c [26]. DPC therefore provides not only a simple and efficient method for growing organic semiconductor single crystals, but also a stable and controllable platform for studying solution crystallization and constructing more complex organic device architectures.

### 2.2. Meniscus-Guided Coating

Meniscus-guided coating (MGC) is a collective term for a range of solution-coating techniques with considerable potential for the large-area and cost-effective fabrication of high-performance organic semiconductor films and organic electronic devices [30]. These methods use the translational motion of either the substrate or the coating tool to induce oriented crystal growth during film deposition. The meniscus is the curved liquid-air interface formed between the coating tool and the substrate. As solvent evaporation drives the solution into supersaturation, crystallization occurs at the three-phase contact line along the meniscus edge, resulting in film formation. During MGC, directional motion of the coating tool drags the meniscus across the substrate, thereby guiding both oriented crystal growth and molecular alignment within the deposited film. MGC is also compatible with industrial roll-to-roll processing and thus offers a promising route toward continuous, large-area fabrication of organic semiconductor films. Accordingly, MGC has become one of the major approaches for preparing organic semiconductor single-crystalline films and has been applied to a wide variety of material systems.

Dip coating is one form of MGC and requires only simple equipment without a dedicated coating tool. As shown in Figure 1d, after the substrate is immersed in the solution, capillary action driven by surface tension causes the liquid to rise along the substrate and form a meniscus. When the substrate is withdrawn upward, pinning at the wetting interface stretches the meniscus, giving rise to films with pronounced anisotropy. Uniform organic semiconductor films can be obtained by appropriately selecting the solvent, solution concentration, and withdrawal speed [27]. For example, Rogowski et al. [31] employed an azeotropic mixed-solvent system together with an optimized withdrawal speed to fabricate oriented ribbon-like TIPS-PEN single-crystal arrays with a mobility of 1 cm^2^ V^−1^ s^−1^. Nevertheless, dip coating requires the substrate to be immersed in a solution reservoir, which consumes relatively large quantities of solution and imposes geometric constraints on the processing space.

Compared with dip coating, blade-based coating requires only a small amount of organic semiconductor solution to be introduced into the gap between the coating tool and the substrate, substantially reducing material consumption. This family of methods includes blade coating, solution shearing, and bar coating. Their deposition processes are broadly similar, with the principal difference being the geometry of the coating tool. Blade coating and solution shearing use blade-shaped tools, typically silicon wafers. In blade coating, the blade is positioned vertically several hundred micrometers above the substrate, whereas in solution shearing, the blade is inclined. Bar coating instead employs a cylindrical coating tool, such as a metal rod. To suppress adsorption of the organic semiconductor, the coating-tool surface is commonly modified with a hydrophobic self-assembled monolayer (SAM) [32]. More elaborate microstructural modifications can also be introduced to improve mass transport. For example, Diao et al. [16] patterned micropillars on the blade surface and controlled their shape and spacing to enhance lateral mass transport perpendicular to the coating direction, thereby reducing void formation and dendritic growth during film deposition. Xiao et al. [33] combined these strategies by modifying patterned micropillars with a superhydrophobic SAM and obtained oriented TIPS-PEN single-crystal arrays by blade coating, with mobilities as high as 6.8 cm^2^ V^−1^ s^−1^. Sheng et al. [17] used solution shearing to prepare monomolecular-layer C8-DNTT films, as shown in Figure 1e, with single-crystalline domain areas reaching 16 mm^2^. OFET arrays containing 95 devices fabricated within a single domain exhibited mobilities of up to 11 cm^2^ V^−1^ s^−1^ and a coefficient of variation (CV) of only 2.57%. Similarly, Zhang et al. [28] employed bar coating, as illustrated in Figure 1f, and optimized the solvent, temperature, and coating speed to produce C8-BTBT films only a few molecular layers thick with millimeter-scale crystalline domains. OFETs fabricated from these films exhibited an average mobility of 13.7 cm^2^ V^−1^ s^−1^ together with excellent device uniformity. These results demonstrate the substantial potential of blade-based coating strategies for fabricating large-area, uniform organic semiconductor single-crystalline films and electronic devices.

### 2.3. Liquid-Surface-Mediated Growth

Liquid-surface-mediated growth is an emerging strategy for fabricating two-dimensional organic semiconductor single crystals. Unlike conventional solution-based approaches, this method employs a liquid surface as the growth substrate. Liquid surfaces are atomically smooth, thereby avoiding the adverse effects of surface defects and roughness associated with conventional solid substrates on crystal nucleation and growth. Selection of an appropriate liquid can substantially promote two-dimensional spreading of the organic semiconductor solution across the liquid surface while suppressing three-dimensional molecular assembly in the vertical direction, ultimately yielding organic single crystals only a few molecular layers, or even a single molecular layer, in thickness.

For example, Xu et al. [18] reported a solution-epitaxy method for obtaining large-area two-dimensional organic semiconductor single crystals on a water surface. As shown in Figure 1g, the process consists of two steps. First, several tens of microliters of organic semiconductor solution are deposited onto water, where the droplets spread under the high surface tension of water. The organic semiconductor molecules aggregate through π-π interactions and evolve into micrometer-scale two-dimensional single crystals, which serve as seeds for the subsequent step. Second, additional solution is supplied to induce epitaxial growth of these two-dimensional seeds, ultimately producing millimeter- to centimeter-scale two-dimensional organic single crystals. Subsequent studies showed that surfactant addition can further promote spreading of the organic semiconductor solution on water and strengthen spatially confined self-assembly, enabling large-area two-dimensional organic single crystals to be obtained in a single step, as shown in Figure 1h [29]. Replacing water with more viscous glycerol can additionally suppress droplet breakup and displacement [34]. The combined use of a surfactant and glycerol therefore maximizes solution spreading at the liquid surface and enables the fabrication of a range of large-area two-dimensional organic semiconductor single crystals.

The fabrication methods discussed above exhibit distinct trade-offs among crystal quality, processing complexity, scalability, and device compatibility. Table 1 therefore provides a qualitative comparison of PVT, DPC, MGC, and liquid-surface-mediated growth in terms of their typical crystal characteristics, potential applications, major advantages, and practical limitations.

This comparison indicates that no single fabrication route is optimal for all applications. The final crystal morphology and properties depend not only on the selected method but also on the regulation of nucleation and growth kinetics. The following section therefore discusses the principal morphological control parameters for organic semiconductor single crystals.

## 3. Morphological Control Strategies for OSSCs

The emergence of large-area and continuous solution-processing techniques has accelerated the application of organic semiconductor single crystals in low-cost, high-throughput, and large-area organic electronics, while the morphology of single-crystalline films remains critically important to device performance [35]. Precise control over film morphology during solution deposition is therefore essential for improving device characteristics. In contrast to typical inorganic semiconductor crystals stabilized by covalent or ionic-covalent bonding networks, small-molecule organic semiconductor single crystals generally consist of intact molecules as their structural units, with lattice stability arising predominantly from noncovalent interactions [36]. Because these intermolecular interactions are strongly directional, cooperative, and competitive, the free-energy differences among alternative molecular packing motifs can be relatively small. Crystal assembly, polymorph selection, and final morphology are therefore highly sensitive to both molecular structure and the crystallization environment [37,38]. Molecular design of organic semiconductors generally prioritizes energy-level alignment, intermolecular electronic coupling, charge-carrier mobility, and multifunctional optoelectronic responses; however, molecular structures favorable for electronic performance do not necessarily satisfy the processing requirements for large-area, ultrathin, uniform, and oriented crystal growth. Consequently, once the thermodynamic tendencies of crystal growth are understood, crystallization kinetics provides a key means of controlling the targeted polymorph, size, thickness, and orientation. Understanding and regulating supersaturation, nucleation rate, mass transport, molecular attachment, and the relative growth rates of different crystal facets can enable more predictive strategies for morphology control and establish a theoretical basis for the controlled fabrication, scalable integration, and device application of organic semiconductor single crystals.

### 3.1. Temperature

**Figure 2:**
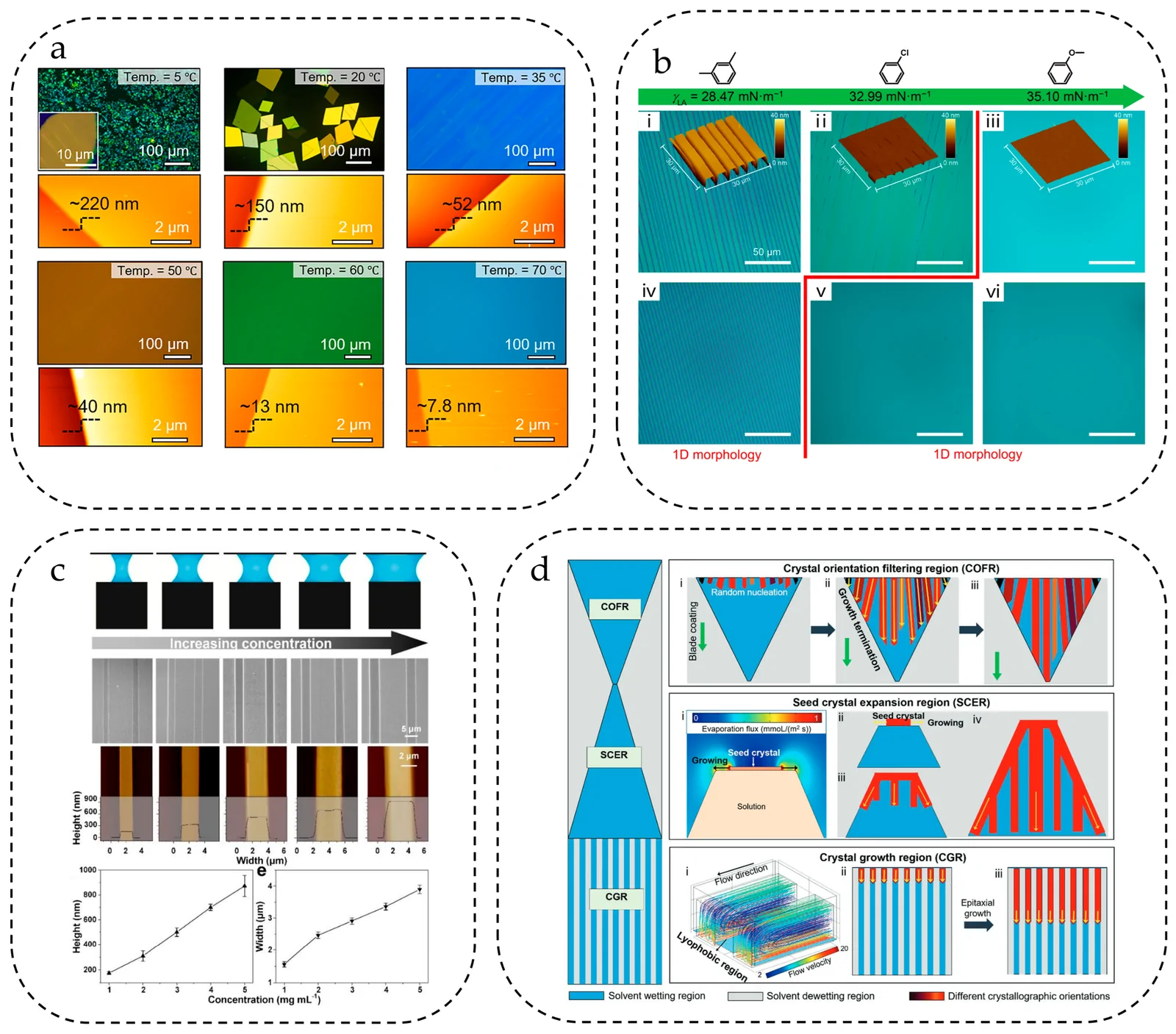
Morphological control of OSSC crystallization kinetics. (**a**) Influence of temperature on the growth of C10-DNTT crystals. Reproduced with permission [40]. Copyright 2025, Wiley-VCH. (**b**) OM images of TIPS-PEN and C8-BTBT crystal morphologies, which were grown from a m-xylene, chlorobenzene, and anisole solution, are presented [43]. (**c**) Mechanism and statistical results of size changing with the solution concentration under a static pressure of 5.5 MPa [45]. (**d**) Illustration of the “orientation filter funnel” concept. Reproduced with permission [46]. Copyright 2022 Wiley-VCH.

Temperature plays a central role in crystallization because it strongly affects supersaturation, nucleation density, and the growth rates of different crystal facets. Changes in crystallization temperature can therefore produce markedly different morphologies even for the same molecule. Fraleoni-Morgera et al. [39] grew 4HCB single crystals by slow solvent evaporation and found that lowering the growth temperature yielded crystals with larger lateral dimensions while preserving a plate-like habit. A lower temperature may slow solvent evaporation and the buildup of supersaturation, reduce secondary nucleation, and prolong the growth time of pre-existing crystals, thereby favoring the formation of larger single crystals. This does not, however, imply that lower temperatures invariably favor large two-dimensional crystals. Wang et al. [40] found that increasing the growth temperature from 35 to 70 °C reduced the average thickness of C10-DNTT single crystals from approximately 75 to 7.8 nm. As shown in Figure 2a, the crystal density decreased substantially, whereas the maximum lateral dimension increased to the millimeter scale. The higher temperature increased material solubility and suppressed the formation of new nuclei, allowing solute to be preferentially supplied to a small number of existing seeds and thereby shifting growth from vertical layer accumulation toward lateral two-dimensional expansion. Liu et al. [41] similarly observed that increasing the growth temperature from 15 to 35 °C gradually transformed TQDPT single crystals from narrow needles into broad plates, with the average width increasing from approximately 2.8 to 30.1 μm. The higher temperature enhanced molecular diffusion and interfacial rearrangement, promoting the growth of previously constrained lateral facets, reducing the crystal aspect ratio, and driving a morphological transition from one-dimensional to two-dimensional growth. Overall, temperature does not regulate crystal morphology simply by accelerating or slowing crystallization; rather, it alters the competition among nucleation, mass transport, and facet-dependent growth rates.

### 3.2. Solvent

Because solution crystallization is a major route for preparing organic semiconductor single crystals, a systematic understanding of how solvents affect crystal growth and morphological evolution is essential. As the medium for solution crystallization, the solvent influences the process through its solvation ability, evaporation rate, surface tension, viscosity, and interactions with the solute. Selective solvent interactions with different crystal facets can further affect crystal size, aspect ratio, thickness, and orientation. For example, Jeong et al. [42] prepared C_60_ microcrystals by antisolvent precipitation using different alcohols as antisolvents and found that simply changing the alcohol produced distinct one-, two-, and three-dimensional crystal morphologies. Crystal size and morphology also varied with the C_60_ concentration and the good-solvent/antisolvent ratio. Differences in the solubility of C_60_ in the various alcohols and in their mixing behavior with the good solvent led to different rates of local supersaturation buildup, nucleation densities, and facet growth rates. Even in single-solvent systems, solvent surface tension can strongly affect crystallization. Ke et al. [43] investigated the effect of solvent surface tension on organic semiconductor crystal morphology using meniscus-guided coating. As the liquid-air surface tension increased, TIPS-PEN evolved from isolated one-dimensional crystalline ribbons into laterally coalesced, continuous two-dimensional crystalline films, as shown in Figure 2b, while the thickness decreased from approximately 25 to 10 nm. This study demonstrated that solvent surface tension can regulate the dimensionality, thickness, and continuity of organic semiconductor crystals by altering interfacial nucleation sites and relative growth rates along different directions. Vladimirov et al. [44] further found that the growth of PDI1MPCN2 in droplets depends not only on solvent surface tension but also on viscosity. High-surface-tension, high-viscosity solvents promoted plate-like growth at the liquid-air interface and produced large-area films, whereas low-surface-tension, low-viscosity solvents yielded discrete three-dimensional bulk crystals. By independently varying viscosity through the addition of polystyrene, the authors showed that neither high surface tension nor high viscosity alone was sufficient to induce crystallization at the liquid-air interface. They proposed that high surface tension provides the principal driving force for heterogeneous nucleation and growth at the interface, whereas high viscosity suppresses Marangoni flow induced by surface-tension gradients, thereby facilitating controlled interfacial crystallization.

### 3.3. Concentration

In solution growth of organic semiconductor single crystals, the initial solution concentration determines how readily the system reaches supersaturation and therefore influences the nucleation density. The concentration also governs the solute flux delivered to the crystal growth front per unit time. Li et al. [47] prepared BPEA single-crystal ribbon arrays using a rapid-annealing-assisted solution-shearing method. When the initial concentration was increased from 0.5 to 1 mg mL^−1^, the thickness of the single-crystal ribbons increased by approximately 10–15 nm; when the concentration was further increased from 0.5 to 2 mg mL^−1^, the density of the ribbon arrays increased markedly. The authors proposed that rapid evaporation generates numerous nuclei at the moving contact line, but at low concentrations only a small fraction of these nuclei receive sufficient solute to sustain continued growth. Increasing the concentration allows more nuclei to exceed the material-supply threshold required for subsequent growth, thereby producing denser and thicker single-crystal ribbon arrays. In systems where nucleation density is constrained, such as template-confined growth, the additional solute supplied at higher concentrations is instead more likely to increase the width and thickness of existing single crystals. Gao et al. [45] confined the nucleation and growth of TIPS-pentacene within nanoscale capillary spaces formed by elastic micropillars. When the precursor concentration was increased from 1 to 5 mg mL^−1^, the resulting single-crystal nanoribbons, shown in Figure 2c, increased in width from approximately 1.6 to 3.9 μm and in thickness from approximately 175.3 to 871.9 nm. Because the confined geometry predetermines the crystal position and growth direction, increasing the concentration primarily increases the amount of solute stored within each capillary space, thereby promoting both lateral and vertical growth of existing crystals. Conversely, when a low concentration is combined with strong confinement, a limited solute supply can lead to incomplete crystallization, surface roughness, or grain-boundary defects.

### 3.4. Substrate

During substrate-assisted crystallization, nuclei generally form preferentially at the solid–liquid interface. By regulating interfacial energy, wettability, chemical interactions, and micro- or nanoscale topography, the substrate can alter the heterogeneous nucleation barrier, three-phase contact-line motion, molecular surface diffusion, and spatial constraints on growth along different crystallographic directions. Rational substrate design has therefore become an important strategy for controlling the position, density, orientation, and morphology of organic semiconductor single crystals.

Chemical functionalization with SAMs provides molecular-scale control over the growth interface without introducing appreciable topographic relief. By varying the terminal groups and spatial patterns of SAMs, the substrate–solution and substrate–crystal interfacial energies can be modified, thereby affecting solution wettability, molecular adsorption and sticking probability, surface diffusion, and heterogeneous nucleation. Importantly, the role of SAMs is not limited to macroscopic wettability. Briseno et al. [48] demonstrated the site-specific growth of anthracene and 5-chlorotetracene single crystals on micropatterned thiol SAMs. The ordered terphenylthiol SAM exhibited orientational and lattice matching with the anthracene (001) plane, suggesting that aromatic interactions and interfacial structural matching promoted oriented nucleation. Li et al. [49] subsequently combined a fluorinated-silane-SAM-based wettability pattern with the selective deposition of a C8-BTBT/PMMA blend and solvent-vapor annealing, resulting in the preferential formation of rod-like C8-BTBT single crystals within predefined channel regions. The importance of terminal-group chemistry was further demonstrated by Pick and Witte [50], who found pronounced differences in the adhesion forces and sticking probabilities of perylene molecules on alkanethiol SAMs with different chemical terminations. Localized liquid-mediated nucleation on a fluorinated SAM additionally produced platelet-shaped perylene single crystallites. These studies show that SAMs can either promote or suppress nucleation and can thereby regulate the spatial distribution and crystallographic orientation of organic semiconductor crystals.

Beyond chemical functionalization, micro- and nanoscale topographic patterning provides a complementary means of controlling liquid confinement, contact-line motion, and crystal orientation. For example, Zhao et al. [51] grew C8-BTBT single crystals on periodic micropillar substrates featuring lyophobic sidewalls and lyophilic top surfaces. Capillary bridges formed between the micropillars partitioned the continuous solution into regular liquid units and induced continuous recession of the three-phase contact line along a prescribed direction, thereby suppressing random dewetting and disordered nucleation. The resulting controlled unidirectional dewetting further guided molecular alignment along a common direction, ultimately producing regularly positioned, uniformly sized one-dimensional single-crystal ribbon arrays with a pure (100) crystallographic orientation. Deng et al. [46] constructed an “orientation-filtering funnel” on the substrate comprising a V-shaped wetting region, a seed-expansion region, and a growth-control region, as schematically illustrated in Figure 2d. Within the initial wetting region, C8-BTBT nuclei with different orientations formed simultaneously, but only nuclei whose growth directions matched the funnel outlet could enter the downstream region; nuclei with other orientations were arrested by the geometric boundaries. The selected seeds then continued to grow, ultimately forming an approximately 1.5 × 1.5 cm^2^ single-crystalline film with highly uniform crystallographic orientation.

## 4. Charge-Transport Mechanisms in OSSCs

Organic semiconductor single crystals are characterized by long-range order and a low density of grain boundaries, and consequently often exhibit relatively low densities of defects and traps. They therefore provide an ideal platform for investigating intrinsic charge-transport behavior and structure-property relationships in organic semiconductors. As discussed above, intermolecular interactions in organic semiconductors are governed primarily by noncovalent forces, and charge transport proceeds through electronic coupling between orbitals on neighboring molecules. At the microscopic level, charge transfer can therefore be described using the transfer integral J and the reorganization energy λ, both of which have dimensions of energy. The transfer integral J describes the electronic coupling between neighboring molecules, with a larger J favoring intermolecular charge delocalization. In contrast, the reorganization energy λ represents the energy associated with molecular-geometry and environmental relaxation during charge transfer, with a larger λ favoring charge localization. Consequently, when J ≥ λ, charge carriers tend to be more delocalized and exhibit band-like transport. When J ≤ λ, carriers are more strongly localized on individual molecules and transport predominantly through thermally activated hopping. When J and λ are comparable, localized and delocalized transport characteristics may coexist [52]. Nevertheless, defects, traps, and dynamic disorder in organic semiconductor single crystals make their charge-transport behavior substantially more complex than that in inorganic crystals. The following sections summarize the principal charge-transport mechanisms in organic semiconductors and representative research progress.

### 4.1. Band-like Transport

Band transport is the classical charge-transport mechanism in inorganic semiconductors. In inorganic crystals, atoms are connected through strong covalent bonds, discrete energy levels broaden into energy bands, and charge carriers propagate through the bands as delocalized Bloch waves. Such transport is particularly susceptible to phonon scattering. In high-purity crystals and over temperature ranges where phonon scattering dominates, increasing temperature generally enhances lattice vibrations and electron-phonon scattering, shortens the carrier relaxation time, and consequently reduces the mobility. Over an appropriate temperature range, this dependence can often be approximated as:

***μ*** = ***A***⋅ ***T***^−***n***^, 0 < ***n*** < 3 (1)

where ***μ*** is the charge-carrier mobility (**cm^2^ V^−1^ s^−1^**), ***A*** is a temperature-independent factor, ***n*** is a dimensionless temperature exponent, and ***T*** is the absolute temperature (K).

**Figure 3:**
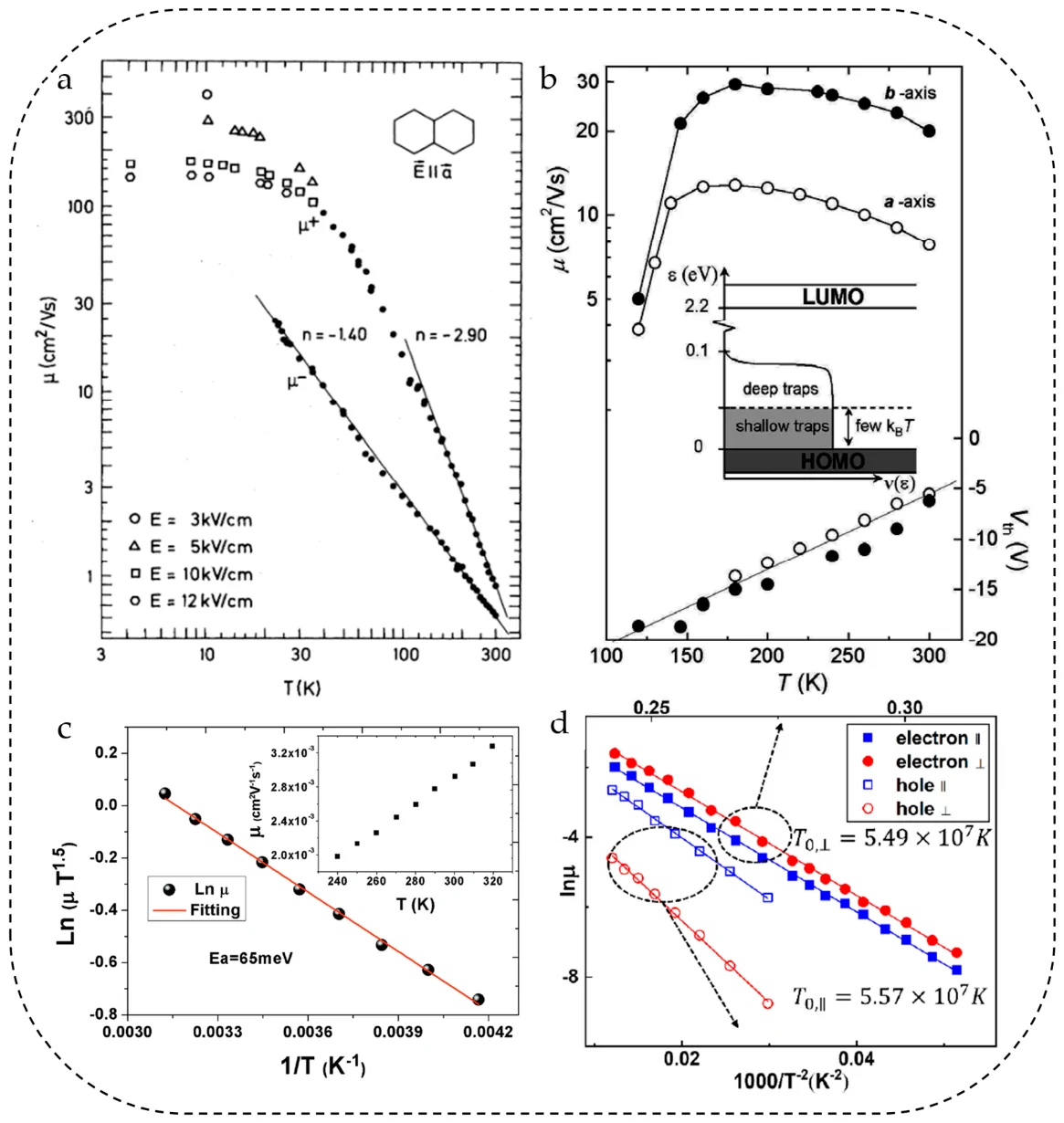
(**a**) Electron and hole mobilities in ultrapure naphthalene. Reproduced with permission [53]. Copyright 1985, American Physical Society. (**b**) The temperature dependence of the field-effect mobility and the threshold voltage measured along the a and b axes of rubrene crystals. Reproduced with permission [57]. Copyright 2004, the American Physical Society. (**c**) TIPS-PEN single crystals: Arrhenius relationship between ln(*μ*_0_*T*^1.5^) and 1/*T*. Reproduced with permission [58]. Copyright 2017, Wiley-VCH. (**d**) Coi8DFIC single crystal: Temperature dependence of hole and electron mobility along the two directions under an electric field of 80 kV/cm. Reproduced with permission [59]. Copyright 2024, American Chemical Society.

In early studies of organic semiconductor single crystals, a negative temperature dependence of carrier mobility was frequently attributed to band transport [6]. For example, high-purity naphthalene single crystals exhibited a pronounced negative power-law dependence of mobility on temperature [53], as shown in Figure 3a. Similar behavior was also observed in crystals such as C_60_ [54] and pentacene [55].

The interpretation of true band transport in organic semiconductors has nevertheless remained controversial. Owing to the distinctive structural characteristics of molecular solids, charge carriers in organic semiconductors are generally delocalized only over a limited spatial range and do not necessarily realize the long-range Bloch-like band transport characteristic of inorganic semiconductors. A negative temperature coefficient of mobility therefore indicates transport with band-like characteristics rather than, by itself, conventional band transport. The study of solution-processed TIPS-PEN thin-film transistors by Sakanoue and Sirringhaus further illustrated this distinction [56]. Spectroscopic measurements of gate-induced charge carriers showed that, at low temperature and low lateral electric field, charges could be localized in shallow trap states on the scale of individual molecules. A moderate increase in lateral electric field promoted detrapping and produced pronounced nonlinear transport at low temperature. Importantly, the negative temperature coefficient of mobility observed at high electric fields was not attributed to conventional transport through extended band states, but was more appropriately interpreted as localized transport limited by thermal lattice fluctuations. As temperature decreases, molecular thermal motion and the associated fluctuations in electronic coupling are reduced, weakening transient localization and thereby increasing carrier mobility.

To account for such behavior, the transient localization model was developed [60,61]. In this framework, strong thermal lattice fluctuations generate dynamic disorder, such that a carrier propagates over a finite distance during a characteristic time τ before undergoing transient localization, thereby limiting carrier diffusion. The corresponding relationship between the diffusion coefficient and mobility can be expressed as:

(2) $$\mu =\frac{eD_{\tau }}{k_{B}T}$$

where ***μ*** is the charge-carrier mobility, ***e*** is the elementary charge, ***D_τ_***  is the transient diffusion coefficient, ***k_B_*** is the Boltzmann constant, ***T*** is the absolute temperature (K), and ***D_τ_*** is given by:

(3) $$D_{\tau }=\frac{L_{\tau }^{2}}{2\tau }$$

where ***L_τ_*** is the transient localization length, namely the finite distance over which a carrier propagates before becoming transiently localized, and ***τ*** is the localization time (s).

Under these conditions, charge transport simultaneously exhibits features of delocalized and localized states. This framework can therefore account for the negative temperature dependence of mobility observed in some high-mobility organic crystals even when the spatial extent of carrier delocalization remains limited.

### 4.2. Thermally Activated Transport

A defining characteristic of the band-like transport discussed above is a negative temperature dependence of carrier mobility. In contrast, transport for which carrier mobility increases with increasing temperature is generally described as thermally activated transport.

#### 4.2.1. Multiple Trapping and Release

Some organic single crystals exhibit band-like transport at relatively high temperatures, yet their carrier mobility does not vary monotonically upon cooling. Instead, the mobility first increases and then decreases. Such behavior has been observed, for example, in rubrene single crystals [57], as shown by the mobility-temperature relationship in Figure 3b. To explain this behavior, the multiple trapping and release (MTR) mechanism, widely used for polycrystalline silicon, has been introduced for organic single crystals [62,63]. In the literature, the temperature dependence is commonly analyzed using the following Arrhenius-type expression:

(4) $$\mu \;=\;\mu_{0}\mathrm{\alpha exp}\left(\frac{-E_{T}}{k_{B}T}\right)$$

Here, *E_T_* is the average activation energy for detrapping from shallow traps, *μ*_0_ is the mobility of carriers in extended states, and *α* is the ratio of the effective density of extended states to that of defect states. In the MTR model, carriers propagating through extended states can be captured by trap states. Carriers captured by deep traps are difficult to release, whereas those captured by shallow traps can be thermally re-excited into extended states and subsequently contribute to transport. Such localized defect states generally originate from impurities, structural disorder, or surface states. The temperature dependence of carrier mobility in rubrene single crystals can therefore be divided into two regimes: transport is dominated by band-like states at high temperatures, whereas MTR becomes dominant at low temperatures. Similar behavior has also been reported in tetracene [64] and PDIF-CN2 [65] single crystals.

#### 4.2.2. Thermally Activated Hopping Transport

When intermolecular electronic coupling is insufficient to sustain extensive carrier delocalization, charge carriers must use thermal lattice vibrations to hop between localized states. The resulting mobility exhibits a positive temperature dependence and is described as thermally activated hopping transport. Common models for such behavior include small-polaron hopping (SPH), variable-range hopping (VRH), and the Gaussian disorder model (GDM).

The small-polaron hopping model [66,67] primarily describes systems in which charge carriers are strongly coupled to intramolecular or lattice vibrations. The presence of a charge can induce local changes in molecular geometry and polarization of the surrounding lattice. When electron-phonon coupling is sufficiently strong, the carrier and the local lattice distortion that it induces can together form a small polaron. The carrier wavefunction is then largely localized on one or a few molecules, and transport proceeds through thermally activated hopping. The mobility associated with small-polaron hopping can generally be expressed as:

(5) $$\mu \;\propto \;T^{-m}\exp\left(\frac{-E_{a}}{k_{B}T}\right)$$

Here, *E_a_* is the effective activation energy for small-polaron hopping, while the exponent m depends on the specific theoretical approximation. In commonly used Holstein-type expressions, *m* = 1 for adiabatic hopping and *m* = 3/2 for nonadiabatic hopping. Wang et al. [58] investigated hole transport along the c-axis of layered TIPS-pentacene single crystals using temperature-dependent space-charge-limited current measurements. The authors fitted the current-voltage characteristics measured at different temperatures and electric fields using a modified SCLC model incorporating small-polaron hopping and extracted parameters including the effective hopping distance, intermolecular transfer integral, and reorganization energy (Figure 3c). The results indicated that, owing to weak electronic coupling perpendicular to the molecular layers, holes are transported predominantly through nearest-neighbor small-polaron hopping between adjacent molecules.

In addition to small-polaron formation, structural disorder can also produce thermally activated hopping transport and can be described using VRH or GDM approaches. VRH applies to disordered systems containing a high density of localized states near the Fermi level. Under these conditions, carriers may hop to sites that are spatially farther from their initial positions while remaining within a narrow energy window near the Fermi level [68,69]. The carrier mobility can be described by Mott’s law:

(6) $$\mu \left(T\right)=\;\mu_{0}\exp\left[-{\left(\frac{T_{0}}{T}\right)}^{p}\right]$$

where *T*_0_ is the characteristic Mott temperature (K) and the exponent *p* takes values of 1/4, 1/3, and 1/2 for three-, two-, and one-dimensional systems, respectively. In the context of organic single crystals, Aleshin et al. [70] investigated PDA-PTS single crystals and found that the low-temperature resistivity could be described by variable-range hopping with Coulomb interactions, whereas transport transitioned to an SPH regime at higher temperatures. This result demonstrates that VRH can emerge even in structurally single-crystalline materials when low dimensionality and localized states play significant roles.

With the development of transport theory, Bässler introduced the Gaussian disorder model to describe carrier transport in disordered solids [71,72]. The model assumes that the energies of localized molecular sites follow a Gaussian distribution and that carriers move among these localized states primarily through phonon-assisted Miller-Abrahams-type hopping. Combined with Monte Carlo simulations, the mobility in the GDM can be expressed as:

(7) $$\mu \left(\sigma ,\Sigma ,T,E\right)=\;\mu_{0}\exp\left[-{\left(\frac{2\sigma }{3k_{B}T}\right)}^{2}\right]\exp\left\{CE^{\frac{1}{2}}\left[{\left(\frac{\sigma }{k_{B}T}\right)}^{2}-\Sigma^{2}\right]\right\}$$

Here, *σ* represents the energetic disorder of the system, whereas *Σ* represents positional disorder, *E* is the magnitude of the applied electric field and *C* is the field-activation coefficient. The model explicitly incorporates the effects of temperature, electric field, carrier concentration, and localized-state density on carrier mobility in a relatively intuitive framework. Although the Gaussian disorder model was originally developed primarily to describe hopping among localized states in amorphous organic semiconductors, energetic and positional disorder can also exist in molecular single crystals with long-range structural order. Pan et al. [59] investigated ambipolar transport in single crystals of the non-fullerene acceptor COi8DFIC using lateral time-of-flight measurements. As illustrated in Figure 3d, the temperature and electric-field dependences of the hole mobility were successfully described by the Gaussian disorder model, revealing different degrees of energetic and positional disorder along directions parallel and perpendicular to crystal growth. In contrast, electron transport in the same single crystal followed a variable-range hopping model and exhibited anisotropic hopping distances.

### 4.3. Identification of Charge-Transport Mechanisms in Organic Semiconductors

As discussed above, the temperature dependence of carrier mobility provides an important experimental basis for identifying transport characteristics in organic semiconductors and can be used to preliminarily distinguish band-like temperature dependence, thermally activated transport, and possible transitions between these regimes. The dependence of carrier mobility on temperature can be summarized as follows [73]:

*lnμ*∝ *T^n^* (8)

The value of the exponent *n* depends on the specific transport model. For band-like transport, *n* is typically in the range of 1–3; for MTR, *n* is commonly −1; for VRH, *n* is typically −0.25; and for GDM, *n* is typically −2.

However, distinct microscopic mechanisms can exhibit similar mobility-temperature dependences over a limited temperature range. Consequently, temperature dependence alone, or agreement with a single empirical fitting model, is generally insufficient to uniquely identify the underlying charge-transport mechanism. A more reliable mechanistic analysis requires multiple complementary forms of evidence, including the electric-field dependence of mobility, Hall-effect measurements, time-of-flight transients, and characterization of defect states.

For example, in the aforementioned study of solution-processed TIPS-PEN thin-film transistors by Sakanoue and Sirringhaus [56], carriers were found to be localized in shallow traps on the scale of individual molecules at low temperature and low lateral electric field. Increasing the lateral field induced carrier detrapping and pronounced nonlinear transport. More importantly, even when the mobility exhibited a negative temperature coefficient at high electric field, spectroscopic evidence showed that transport did not correspond to conventional band conduction through extended states, but rather to localized transport limited by thermal lattice fluctuations. This study directly demonstrates that μ-T relationships must be interpreted together with electric-field dependence and spectroscopic evidence.

The Hall effect is also widely used to probe charge transport in organic semiconductors. Podzorov et al. [9] measured the Hall effect in rubrene single-crystal OFETs and found that, in the intrinsic transport regime at relatively high temperatures, the Hall carrier density *n_H* was approximately consistent with the induced charge density calculated from the gate capacitance. In the trap-dominated low-temperature regime, by contrast, *n_H* was lower than the total induced charge density, indicating that a fraction of the carriers was localized in traps. Comparing Hall mobility and Hall carrier density with field-effect mobility therefore provides insight into the effects of trapping and localization.

Time-of-flight (TOF) measurements are likewise widely used to characterize carrier mobility in organic semiconductors. In experiments on tetracene, De Boer et al. [64] showed that shallow traps affect both the effective mobility measured by TOF and its temperature dependence, whereas deep traps primarily reduce the number of carriers that reach the counter electrode. By analyzing the transient response and its temperature dependence, the authors estimated characteristic trap energies and relative trap concentrations, demonstrating that TOF provides not only the carrier transit time but also information on trapping, release, and transport dispersion.

## 5. Electronic and Optoelectronic Devices Based on OSSCs

Before discussing specific device types, it is useful to compare amorphous and single-crystalline organic semiconductors from a practical perspective. Amorphous and structurally disordered organic semiconductors can be deposited as continuous films and readily incorporated into multilayer or doped structures. These processing advantages have enabled their extensive use in OLEDs, OPDs, and thin-film organic lasers [74,75,76]. However, disordered molecular packing can introduce energetic disorder and trap states, leading to dispersive charge transport, reduced carrier mobility, and slower device response [77]. In contrast, organic single crystals generally exhibit fewer defects, higher carrier mobility, and well-defined optical anisotropy, offering advantages for high-performance transistors, low-noise or polarization-sensitive photodetectors, polarized light-emitting devices, and low-loss lasers. Their practical application is nevertheless limited by difficulties in controlling crystal dimensions, forming reliable electrical contacts, and integrating crystals into large-area device arrays. Therefore, amorphous materials offer greater manufacturing flexibility, whereas single crystals provide higher intrinsic performance in selected applications. The following sections discuss these characteristics in different electronic and optoelectronic devices.

Electronic and optoelectronic devices based on organic single crystals have undergone rapid development over the past decade. These devices can be broadly classified into organic field-effect transistors and logic circuits, organic photoelectric conversion devices, and organic electroluminescent devices. The following sections review recent progress in these major categories of devices based on organic semiconductor single crystals.

### 5.1. Organic Field-Effect Transistors and Organic Circuits

Organic semiconductor single-crystal field-effect transistors are not only important platforms for investigating intrinsic carrier transport in organic semiconductors, but also fundamental device elements for constructing organic electronic circuits [78]. Their long-range structural order, low grain-boundary density, and well-defined crystallographic orientation enable high mobility and stable switching characteristics. In integrated circuits comprising multiple OFETs, however, variations in crystal size, thickness, orientation, and electrode contact can lead to substantial device-to-device dispersion in mobility, threshold voltage, contact resistance, on/off ratio, and other parameters [17]. Consequently, research on organic semiconductor single-crystal OFETs is progressively shifting toward the controlled fabrication of large-area, oriented, and patterned single-crystal arrays, improved uniformity of device parameters, and the construction of basic circuit elements such as inverters.

**Figure 4:**
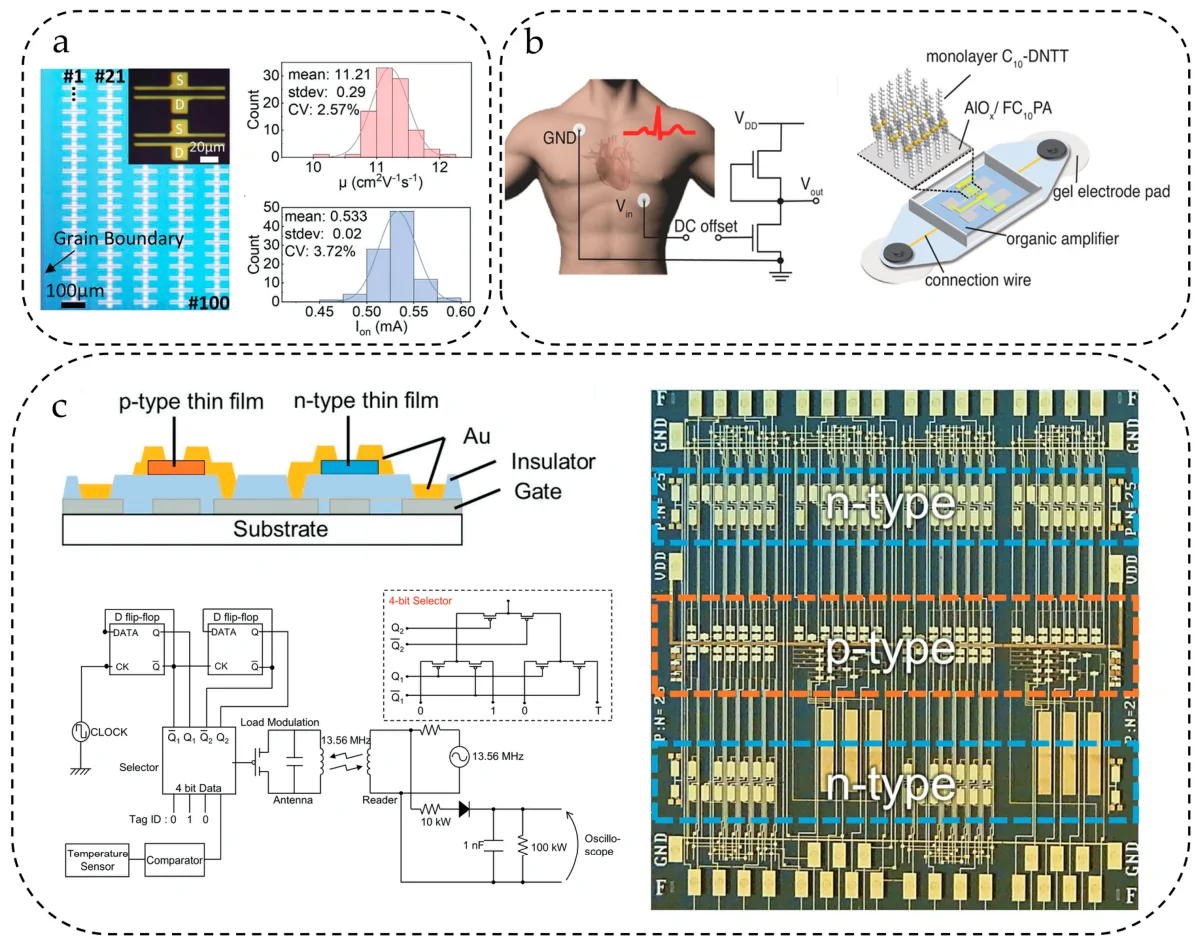
(**a**) The POM image of the 20 × 5 OFET array and the histograms of mobility. Reproduced with permission [17]. Copyright 2023, Wiley-VCH. (**b**) Flexible laminated organic inverter for ECG application. Reproduced with permission [80]. Copyright 2022, Wiley-VCH. (**c**) A photographic image of the CMOS device. (**Left**) A schematic of the RFID prototype and (**right**) a circuit diagram of the 4-bit selector. Reproduced with permission [81]. Copyright 2017, Wiley-VCH.

Large-area single-crystal arrays with uniform orientation and controlled thickness constitute a fundamental materials platform for circuit-level integration of organic semiconductor single crystals. Wang et al. [79] employed an anchored-seed epitaxial-growth strategy to obtain wafer-scale, few-layer diF-TES-ADT single crystals on the surface of a viscous liquid. Continuous growth from the same seed effectively reduced crystallographic and structural variations across different regions. The resulting OFETs exhibited mobilities of up to 8.6 cm^2^ V^−1^ s^−1^, with the mobility CV reduced to 8.9%. These results indicate that simply maximizing crystal size is not the sole objective of single-crystal integration; simultaneous control over nucleation position, growth direction, and crystal thickness is more important for achieving stable device characteristics. Sheng et al. [17] further addressed OFET uniformity by using layer-number-controlled, single-domain molecular-monolayer C8-DNTT crystals as the channel. The devices exhibited narrowly distributed contact resistance (79.00 ± 7.00 Ω·cm) and intrinsic mobility (12.36 ± 0.45 cm^2^ V^−1^ s^−1^), with a mobility CV of only 2.57%, as shown in Figure 4a. Such studies have contributed to a shift in OFET evaluation from record mobility toward average performance, parameter distributions, device yield, and operational stability.

Once high-performance and uniform single-crystal OFET arrays are available, devices with a single carrier polarity can be further used to construct unipolar circuits. Such circuits generally employ only p-type or n-type OFETs and realize logic inversion through diode-connected loads, depletion-mode loads, or pseudo-CMOS architectures composed of multiple transistors of the same polarity. Duan et al. [82] constructed pseudo-CMOS inverters and NAND logic gates using p-type single-crystal OFET arrays, demonstrating that unipolar single-crystal devices can support both inversion and combinational logic functions. He et al. [80] used solution-grown organic monolayer single crystals together with van der Waals electrode assembly to construct high-gain inverting amplifiers and applied them to the acquisition of weak electrocardiographic signals, as shown in Figure 4b. This work illustrates the evolution of unipolar single-crystal circuits from basic logic demonstrations toward sensor-signal amplification and readout. A principal advantage of unipolar circuits is that only the growth, patterning, and electrical contacting of one semiconductor polarity need to be addressed. However, because the load and driving transistors can conduct simultaneously under certain operating conditions, these circuits often suffer from relatively high static power consumption, incomplete output swing, and limited noise margins.

Complementary inverters provide an alternative to unipolar organic circuits and are attractive because of their low power consumption, compact footprint, and reduced transistor count for a given logic function. A complementary inverter can be implemented using separate unipolar n-type and p-type OFETs or a single ambipolar OFET. In 2006, Briseno et al. [83] constructed the first organic single-crystal complementary inverter using a manual transfer strategy, although the gain was limited by the mismatch between carrier mobilities in the p-type and n-type semiconductors. Subsequent studies achieved higher inverter gains by appropriately pairing p-type and n-type semiconductors, such as C60/TIPS-PEN [84] and diF-TES-ADT/TU-3 [85], to obtain more balanced carrier mobilities and low densities of shallow defects. Takeya et al. [81] advanced this concept toward greater system integration by fabricating millimeter-scale, highly oriented p-type and n-type single-crystalline films over large areas on the same substrate. These films were used to construct complementary logic circuits and were further integrated with a temperature sensor, 4-bit digital encoding, and a near-field wireless communication module to realize a prototype RFID sensor tag operating at 13.56 MHz, as shown in Figure 4c. Inverters can also be implemented using ambipolar transistors [86] with a simpler fabrication flow, although this approach relies primarily on the development of suitable ambipolar organic semiconductors and is not discussed in detail here.

In addition to separately preparing p-type and n-type single crystals, constructing single-crystal p-n heterojunctions with parallel electron and hole transport channels provides another important route toward ambipolar circuits. For example, Li et al. [26] used droplet-pinned crystallization to fabricate a TIPS-TAP/TIPS-BP single-crystal heterojunction in a single step. The device exhibited relatively balanced electron and hole mobilities of 1.9 and 1.02 cm^2^ V^−1^ s^−1^, respectively. An inverter based on this structure showed a sharp transition near 30 V at a supply voltage of 60 V and achieved a voltage gain of 70. Unlike conventional complementary circuits composed of separate p-type and n-type OFETs, a p-n single-crystal heterojunction integrates both carrier channels and a structurally well-defined donor-acceptor interface within a single device. This architecture not only enables logic operation but also provides a unified heterointerface platform for constructing other organic semiconductor optoelectronic devices.

### 5.2. Organic Photoelectric Conversion Devices

The operation of organic semiconductor photoelectric conversion devices can generally be described in terms of light absorption, exciton diffusion and dissociation, followed by the transport and collection of free charge carriers. Organic semiconductor single crystals, with their long-range order, low grain-boundary density, and well-defined molecular orientation, are advantageous for reducing transport disorder and extending exciton diffusion lengths. However, organic semiconductors generally exhibit relatively high exciton binding energies; therefore, constructing efficient exciton-dissociation interfaces and local electric fields is a key strategy for single-crystal photoelectric conversion devices [5,87]. The following discussion focuses on two representative device classes based on organic semiconductor single crystals: organic phototransistors and organic photovoltaics.

**Figure 5:**
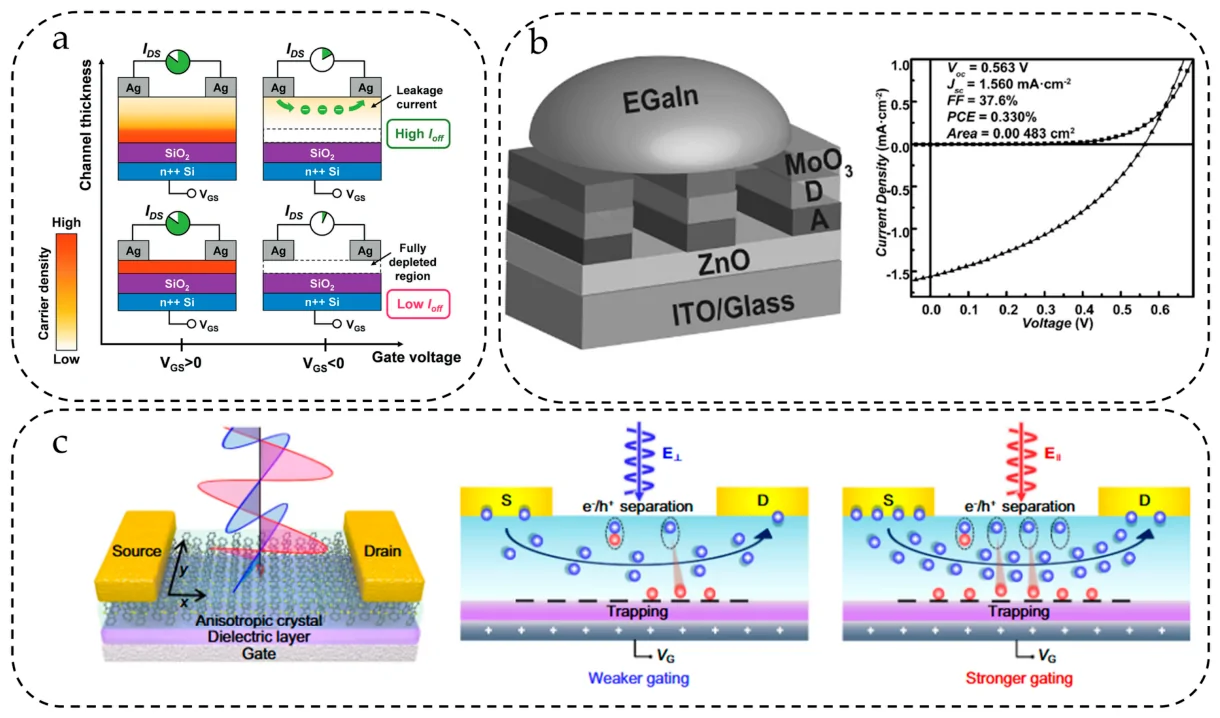
(**a**) Schematic diagram of two devices with different thicknesses operated under dark conditions. Reproduced with permission [89]. Copyright 2018, 2018 Wiley-VCH. (**b**) Solar cell performance of single-crystalline donor-acceptor heterojunctions Reproduced with permission [93]. Copyright 2015, Wiley-VCH. (**c**) Anisotropic charge trapping effect for polarization sensitivity enhancement in phototransistors [94].

Single-component organic single-crystal photoelectric devices are most commonly implemented as organic phototransistors. Photoexcitation can directly alter the carrier concentration in the channel and generate mobile charge carriers. When one type of photogenerated carrier produced after exciton dissociation is trapped for an extended period by defects or interfaces, the remaining carriers can continuously modulate the channel potential through a photogating effect. The difference between carrier lifetime and transit time can then produce substantial photoconductive gain. Jones et al. [88] placed a rubrene single crystal on top of a graphene transistor, with the rubrene serving as the visible-light absorber and long-range exciton-transport medium. Following exciton dissociation at the rubrene-graphene interface, photogenerated holes were transferred into graphene for rapid transport, while electrons remained in the rubrene crystal and continuously photogated the graphene channel, resulting in pronounced photoconductive gain and high responsivity. Subsequent reductions in crystal dimensionality and optimization of device architecture further expanded the functionality of single-crystal phototransistors. Wang et al. [89] prepared n-type TFT-CN two-dimensional single crystals thinner than 5 nm, which exhibited strong absorption near 830 nm and were used to construct near-infrared phototransistors. As shown in Figure 5a, the ultrathin single crystals exhibited an exceptionally low off-state current in the dark compared with thicker crystals, highlighting the advantages of combining low-dimensional single crystals with n-type near-infrared-responsive materials. Liu et al. [90] constructed organic single-crystal vertical field-effect transistors and phototransistors using a 2,6-diphenylanthracene (DPA) single crystal as the vertical active layer and graphene as a tunable source electrode. The vertical architecture shortened the effective carrier-transport distance, while illumination and gate-voltage modulation of the graphene-DPA Schottky barrier enabled high current density together with effective suppression of dark current. Cao et al. [91] further found that p-MSB single crystals exhibited substantially enhanced photoresponses as their thickness approached the two-dimensional limit, indicating that crystal thinning can not only shorten the transport distance but may also regulate photogenerated charge behavior through changes in molecular packing and interfacial effects. Beyond packing and device-architecture engineering, Tao et al. [92] designed DTEP-derivative single crystals for ultraviolet detection that combined relatively high mobility, strong ultraviolet absorption, and a low exciton binding energy, highlighting the importance of synergistically optimizing molecular structure and crystal packing.

Exciton dissociation is intrinsically limited in single-component organic single crystals, whereas constructing organic single-crystal p-n heterojunctions provides an effective strategy for facilitating charge separation. This requirement is particularly important for organic photovoltaics, in which exciton dissociation, free-carrier separation, and charge collection must proceed continuously under the built-in electric field. The formation of single-crystal p-n heterojunctions is therefore central to realizing organic single-crystal photovoltaic devices. Li et al. [93] directly grew single-crystalline donor-acceptor heterojunctions with a stable vertical stacking sequence from a mixed solution containing a C60 acceptor and a DPP-based donor. The resulting photovoltaic devices, whose structure and performance are shown in Figure 5b, achieved an average power conversion efficiency of 0.255 ± 0.095%. Although this efficiency was substantially lower than that of contemporary thin-film organic solar cells, the study provided the first demonstration that single-crystalline donor-acceptor photovoltaic junctions could be fabricated in batches through solution processing, advancing organic single crystals from model materials for studying exciton and charge transport toward practical photovoltaic active layers. In subsequent work, Xiao et al. [95] vertically stacked p-type C6-DPA and n-type NDI molecular-monolayer crystals to construct a single-crystal p-n heterojunction with a total thickness below 5 nm. The well-defined donor-acceptor interface supported balanced ambipolar transport and also produced a gate-tunable photovoltaic response, demonstrating that molecular-layer single-crystal heterojunctions can simultaneously enable photodetection, photovoltaic conversion, and electrical modulation.

Organic single crystals also possess pronounced anisotropy, leading to crystallographic-direction-dependent light absorption and charge transport and making them particularly suitable for polarized-light detection. Pan et al. [94] exploited differences in optical absorption and carrier trapping along different crystallographic axes of C8-BTBT single crystals to construct phototransistors with strong polarization selectivity, as schematically illustrated in Figure 5c. Based on this mechanism, the devices achieved high polarization discrimination and were further used to emulate biological perception of skylight polarization, enabling directional recognition and biomimetic navigation. These results demonstrate that organic single-crystal optoelectronic devices are evolving beyond simple optical-signal detection toward intelligent optoelectronic systems capable of information extraction and environmental perception.

### 5.3. Organic Light-Emitting and Laser Devices

Organic semiconductor single-crystal electroluminescent devices operate through the injection of electrons and holes from electrodes, followed by carrier transport and recombination within the ordered crystal to form excitons, whose radiative decay converts electrical energy into light. Compared with amorphous films, single crystals possess fewer grain boundaries, lower trap densities, well-defined crystallographic orientations, and higher carrier mobilities, all of which help reduce transport losses and can enable polarized light emission. At the same time, close molecular packing can promote fluorescence quenching and nonradiative recombination, requiring molecular design to balance efficient charge transport with strong emission [96]. At the device level, organic single-crystal electroluminescent devices can be broadly divided, according to their carrier-injection geometry and control of the recombination zone, into vertically structured single-crystal OLEDs and laterally configured, gate-controlled single-crystal organic light-emitting transistors (OLETs).

**Figure 6:**
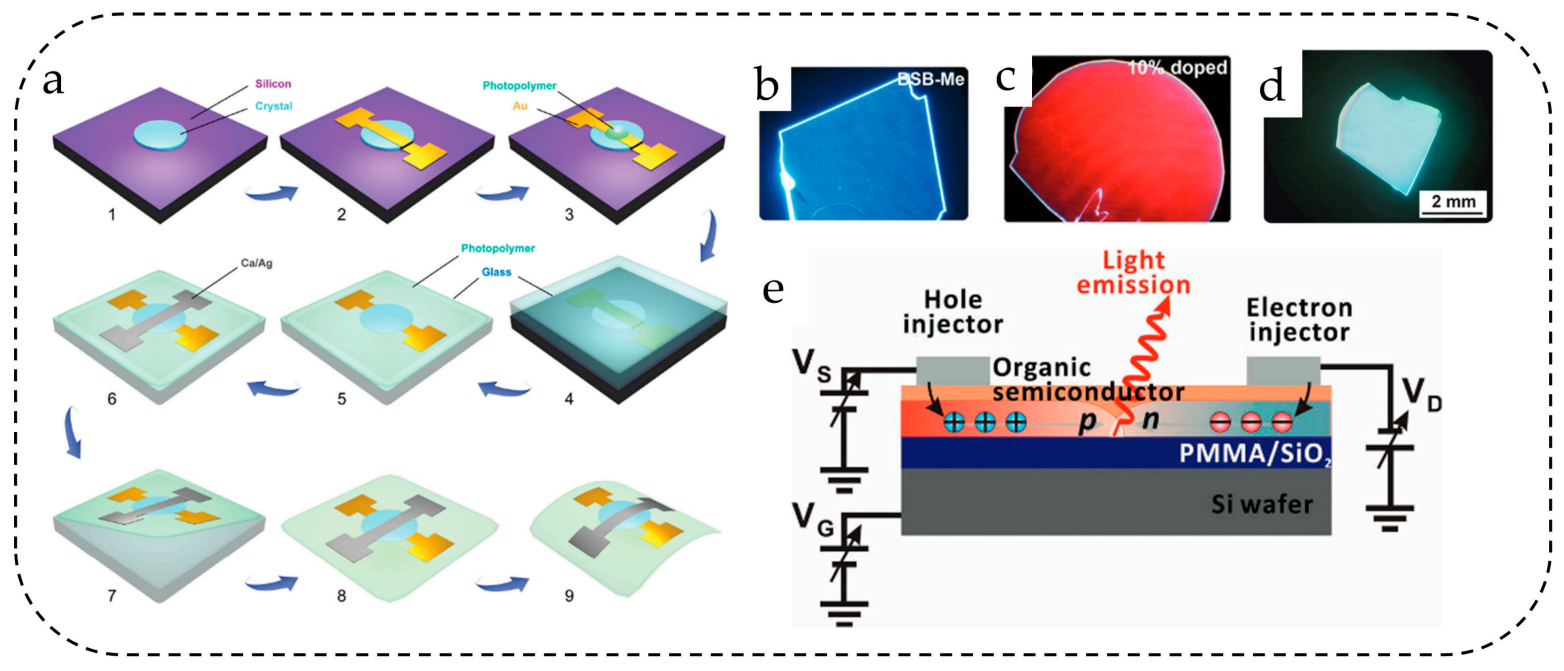
(**a**) Schematic of fabrication process for crystal-based OLEDs based on the template stripping technique. Reproduced with permission [97]. Copyright 2014 Wiley-VCH. (**b**,**c**) Optical photograph of the pure crystals and the doped crystals. Reproduced with permission [99]. Copyright 2018 Wiley-VCH. (**d**) Optical photograph of the double-doped crystal under UV light. Reproduced with permission [100]. Copyright 2018 Wiley-VCH. (**e**) An operational schematic of ambipolar light-emitting transistors. Reproduced with permission [103]. Copyright 2011 Wiley-VCH.

As organic single-crystal OLEDs progressed from proof-of-concept demonstrations toward performance optimization, considerable effort focused on improving carrier injection and controlling emission color. Ding and co-workers made a series of contributions in these areas, as illustrated in Figure 6a–d. They employed a template-stripping method to improve the contacts between the upper and lower metal electrodes and the single-crystal surfaces, thereby increasing the efficiency and spatial uniformity of carrier injection, suppressing localized current injection caused by poor contact, and producing more uniform electroluminescence [97]. They subsequently introduced hole-buffer and electron-transport layers between the crystal and the electrodes to improve the balance of electron and hole injection and transport, enabling red, green, and blue primary-color single-crystal light-emitting devices [98]. At the materials level, they further explored host-guest molecular doping and showed that emission color could be tuned through energy transfer while largely preserving lattice order. The use of a dual-guest doping strategy further enabled white-light single-crystal devices with a high color-rendering index [99,100]. More recently, the well-defined orientation of transition dipoles in single crystals has been exploited to construct intrinsically linearly polarized LEDs. Jia et al. [101] used the highly ordered molecular arrangement and transition dipole moments preferentially aligned along specific crystallographic axes in organic single crystals to fabricate intrinsically linearly polarized light-emitting diodes based on DPA and BSB-Me single crystals. Fu et al. [102] further integrated an organic single-crystal emissive layer into an optical microcavity and tuned the cavity modes and photonic spin–orbit coupling to realize direction-dependent circularly polarized electroluminescence, avoiding the conventional requirement for exclusively chiral emissive molecules. These studies demonstrate the evolution of single-crystal OLEDs from improved electrode contacting toward full-color, white-light, and polarized emission. Nevertheless, their further development remains constrained by crystal-thickness control, out-of-plane charge transport, damage during top-electrode fabrication, and large-area manufacturing.

In comparison, OLETs can more fully exploit the high in-plane mobility of organic single crystals. Electrons and holes are injected from the source and drain electrodes, respectively, and transported along the crystal/dielectric interface under the gate field before recombining. By adjusting the gate and source-drain voltages, the position and width of the emission zone can be controlled, allowing the exciton-recombination region to be displaced away from the metal electrodes and thereby reducing electrode-induced exciton quenching. Takahashi et al. [104] first demonstrated ambipolar transport and channel electroluminescence in tetracene single crystals. Bisri et al. [105] subsequently improved the balance between electron and hole transport and showed that the emissive region in the channel can be regarded as a gate-field-induced p-i-n homojunction. With the emergence of materials that combine high mobility with strong solid-state emission, a single crystal in a single-component OLET can simultaneously support electron transport, hole transport, and exciton recombination, leading to improved external quantum efficiency. The single crystals with balanced ambipolar transport increased the external quantum efficiency of OLETs to 20.5%; ref. [106] demonstrated the progression of single-crystal OLETs from proof of electroluminescence toward highly efficient devices.

Beyond electrically driven emission, OSSCs also exhibit distinctive photoluminescence (PL) and stimulated-emission properties. Although close molecular packing can induce fluorescence quenching, rational control of molecular structure and crystal packing can preserve efficient radiative decay while maintaining ordered charge transport. For example, TPA-An and TBA-An ribbon crystals exhibited room-temperature PL quantum yields of 98% and 99%, respectively, together with effective hole transport, demonstrating that strong solid-state emission and semiconducting behavior can coexist in a single crystal [107]. Their well-defined transition-dipole orientations can additionally produce anisotropic and polarized PL, while smooth surfaces and regular crystal facets facilitate optical waveguiding.

These characteristics make OSSCs promising gain media for compact organic lasers. Single crystals can provide both optical gain and waveguiding, while their natural facets or external gratings can supply optical feedback. Ma et al. demonstrated efficient distributed-feedback lasing by placing three types of organic single crystals on patterned gratings [108]. DBTVB single-crystal microplates further combined high PL efficiency, balanced ambipolar transport, efficient OLET operation, and low-threshold optical lasing [109]. More recently, single-crystal polariton OLEDs have linked high-brightness electroluminescence with optically pumped polariton lasing [110], and room-temperature continuous-wave lasing has been achieved using a monolayer molecular crystal as the gain medium in an hBN microdisk [111]. Nevertheless, most OSSC lasers still rely on optical pumping because direct electrical pumping requires high current densities together with effective control of charge balance, triplet-related losses, and thermal degradation. OSSC lasers therefore show considerable potential for polarized microscale light sources, optical sensing, integrated photonics, and laser displays, whereas electrically driven operation remains an important longer-term objective.

## 6. Summary and Outlook

This review systematically summarizes research progress in organic semiconductor single crystals, with emphasis on controlled fabrication, charge transport, and device applications. Representative continuous-fabrication strategies, including droplet-pinned crystallization, meniscus-guided coating, and liquid-surface-mediated growth, were first discussed, highlighting the evolution of organic single crystals from discrete bulk crystals toward large-area, ultrathin, oriented, and patterned single-crystal arrays. The effects of external conditions, including temperature, solvent, solution concentration, and substrate, on nucleation behavior, mass transport, and facet-dependent growth rates were then examined from the perspective of crystallization kinetics, demonstrating that crystal size, thickness, orientation, and morphology arise from the combined influence of multiple thermodynamic and kinetic factors. On this basis, the review further discussed charge-transport models including band-like transport, multiple trapping and release, small-polaron hopping, variable-range hopping, and Gaussian disorder, while emphasizing that microscopic transport mechanisms should be identified by integrating temperature and electric-field dependences with Hall-effect measurements, time-of-flight transients, and defect-state characterization. Finally, applications of organic single crystals in field-effect transistors, logic circuits, photodetectors, photovoltaics, and electroluminescent devices were summarized, showing that the research focus is progressively shifting from maximizing the performance of individual devices toward device arrays, functional integration, and complex optoelectronic systems.

Despite substantial progress in organic semiconductor single crystals, the field continues to face fundamental scientific challenges. First, a broadly applicable predictive theory of single-crystal crystallization remains lacking. Temperature, supersaturation, solvent evaporation rate, substrate wettability, and solvent-solute interactions often vary simultaneously [16] and collectively determine nucleation position, polymorph selection, and growth rates along different crystallographic directions. Consequently, the same processing parameter can produce entirely different morphological evolution in different material or solvent systems, and many current studies still rely heavily on empirical screening of growth conditions. Future work should make greater use of in situ optical microscopy, in situ X-ray scattering, vibrational spectroscopy, and related techniques to monitor nucleation, polymorphic transitions, and growth-front evolution in real time. These measurements should be combined with molecular dynamics, fluid dynamics, and phase-field modeling to connect intermolecular interactions and interfacial nucleation with macroscopic mass transport. Building high-throughput experimental and machine-learning databases linking molecular structure, crystallization conditions, crystal structure, and morphology may ultimately enable target-oriented design of organic single-crystal growth with a prescribed polymorph, thickness, and orientation.

Second, unified and reliable criteria for identifying charge-transport mechanisms in organic semiconductor single crystals remain to be established. Long-range structural order does not preclude carrier localization or hopping transport. Molecular thermal motion can cause temporal fluctuations in transfer integrals and introduce dynamic disorder, whereas impurities, residual solvents, point defects, lattice strain, and interfacial polarization can generate static disorder or localized traps. Consequently, band-like transport, transient localization, multiple trapping and release, and thermally activated hopping may interconvert or coexist under different temperatures, electric fields, carrier concentrations, or crystallographic directions [56,112]. An increase in mobility upon cooling can therefore serve only as preliminary evidence of band-like behavior and cannot, by itself, establish conventional long-range extended-state transport. Similarly, a single Arrhenius or variable-range-hopping fit cannot uniquely identify the microscopic mechanism. Future studies should establish multidimensional experimental criteria spanning temperature, electric field, carrier concentration, crystallographic direction, and timescale, and should combine Hall-effect measurements, time-of-flight methods, terahertz spectroscopy, and sub-bandgap defect-state characterization.

Beyond these fundamental questions, the practical and industrial deployment of organic semiconductor single crystals remains constrained by scalable manufacturing, device uniformity, and long-term reliability [113,114]. Future evaluation should therefore shift from emphasizing the largest crystal size and highest mobility toward metrics such as crystal coverage, thickness distribution, crystallographic-orientation uniformity, device yield, coefficient of variation of key parameters, and batch-to-batch reproducibility. Despite their low grain-boundary density, organic semiconductor single crystals and the corresponding devices are not intrinsically immune to environmental degradation. In pentacene single crystals, oxygen can reversibly increase the hole concentration through acceptor-like doping, whereas water incorporated into the crystal can generate charge-trapping sites [115]. At the device level, water adsorbed at the semiconductor/dielectric interface can form discrete trap states under electrical bias, perturb interfacial charge accumulation, and consequently cause mobility degradation, threshold-voltage shifts, and hysteresis [116]. Rubrene single crystals are relatively resistant to oxygen in the dark; however, simultaneous exposure to light and oxygen initiates photo-oxidation at molecular step edges and surface defects and subsequently promotes oxygen penetration into the crystal [117]. Therefore, dark storage stability should be distinguished from operational stability under illumination and electrical bias. Practical protection strategies include processing and storing crystals under dry or inert conditions, limiting prolonged illumination, and encapsulating the complete device with conformal barrier layers. Parylene encapsulation, for example, can suppress moisture-related trap formation and improve mobility and threshold-voltage stability during operation in ambient air, although the permeability of the encapsulant should be matched to the dominant degradation pathway of the specific semiconductor and device structure [118].

In addition, constructing multicomponent systems based on organic semiconductor single crystals, including single-crystal heterojunctions [26] and co-crystals [119], provides an alternative and promising strategy for integrating multiple functions, enhancing optoelectronic performance, and enabling new functionality. Nevertheless, forming heterostructures with controlled integration across both macroscopic and molecular length scales, while simultaneously combining or enhancing multiple functions, remains challenging.

Overall, future progress in organic semiconductor single crystals will depend on the development of predictive crystallization theories, reliable criteria for identifying charge-transport mechanisms, and device technologies compatible with scalable processing. Advances in these areas should allow organic semiconductor single crystals to further exploit their distinctive advantages in fundamental physical studies, high-performance flexible electronics, intelligent optoelectronic sensing, and integrated optoelectronic systems.

## Figures and Tables

**Table 1:** Comparison of representative fabrication methods for OSCs.

Fabrication Method	Crystal Quality	Fabrication Complexity and Scalability	Potential Applications	Main Advantages	Main Limitations
Physical vapor transport (PVT)	High-purity, highly ordered bulk or platelet single crystals with low defect densities.	Relatively complex and time-consuming; mainly suitable for batch preparation of discrete crystals, with limited scalability.	Intrinsic-property studies, spectroscopy, crystallography and benchmark OFETs.	Simultaneous purification and crystallization; excellent structural order; negligible solvent residue.	Elevated temperature and specialized equipment; limited control over crystal position, thickness and orientation; difficult large-area integration.
Droplet-pinned crystallization (DPC)	Aligned ribbon- or needle-like single-crystal arrays with relatively high crystallinity.	Simple equipment and low processing cost; extendable to large areas using multiple pinners, although uniformity remains challenging.	Aligned OFET arrays, patterned devices and single-crystal heterojunctions.	Low-temperature processing; directional and positional growth; compatible with one-step heterojunction formation.	Sensitive to evaporation, droplet volume and substrate wettability; thickness and coverage may be nonuniform.
Meniscus-guided coating (MGC)	Oriented single-crystalline films or large domains with controllable thickness and good uniformity.	Moderate process complexity; highly compatible with continuous and roll-to-roll manufacturing.	Large-area OFET arrays, integrated circuits, sensors and flexible electronics.	Continuous deposition; good control over orientation and thickness; high throughput and material utilization.	Narrow processing window; meniscus instability may produce voids, grain boundaries and thickness variations.
Liquid-surface-mediated growth	Large-area, monolayer or few-layer two-dimensional single crystals with low substrate-induced disorder.	Moderate complexity; large-area growth is feasible, but transfer and device integration limit current scalability.	Ultrathin OFETs, flexible devices and transferable or van der Waals structures.	Atomically smooth growth interface; suppresses vertical aggregation; enables large-area ultrathin crystals.	Restricted material–solvent combinations; transfer may introduce contamination, wrinkles or cracks; positioning remains difficult.

## Data Availability

No new data were created or analyzed in this study. Data sharing is not applicable to this article.
